# Using *in silico* tools to predict flame retardant metabolites for more informative exposomics-based approaches

**DOI:** 10.3389/ftox.2023.1216802

**Published:** 2023-10-16

**Authors:** Breanne Kincaid, Przemyslaw Piechota, Emily Golden, Mikhail Maertens, Thomas Hartung, Alexandra Maertens

**Affiliations:** ^1^ Center for Alternatives to Animal Testing (CAAT), Johns Hopkins Bloomberg School of Public Health, Baltimore, MD, United States; ^2^ CAAT-Europe, University of Konstanz, Konstanz, Germany

**Keywords:** in silico spectra, metabolite prediction, exposomics, flame retardant (FR), metabolomics, hazard assessment, risk assessment, chemical classification

## Abstract

**Introduction:** The positive identification of xenobiotics and their metabolites in human biosamples is an integral aspect of exposomics research, yet challenges in compound annotation and identification continue to limit the feasibility of comprehensive identification of total chemical exposure. Nonetheless, the adoption of *in silico* tools such as metabolite prediction software, QSAR-ready structural conversion workflows, and molecular standards databases can aid in identifying novel compounds in untargeted mass spectral investigations, permitting the assessment of a more expansive pool of compounds for human health hazard. This strategy is particularly applicable when it comes to flame retardant chemicals. The population is ubiquitously exposed to flame retardants, and evidence implicates some of these compounds as developmental neurotoxicants, endocrine disruptors, reproductive toxicants, immunotoxicants, and carcinogens. However, many flame retardants are poorly characterized, have not been linked to a definitive mode of toxic action, and are known to share metabolic breakdown products which may themselves harbor toxicity. As U.S. regulatory bodies begin to pursue a subclass- based risk assessment of organohalogen flame retardants, little consideration has been paid to the role of potentially toxic metabolites, or to expanding the identification of parent flame retardants and their metabolic breakdown products in human biosamples to better inform the human health hazards imposed by these compounds.

**Methods:** The purpose of this study is to utilize publicly available *in silico* tools to 1) characterize the structural and metabolic fates of proposed flame retardant classes, 2) predict first pass metabolites, 3) ascertain whether metabolic products segregate among parent flame retardant classification patterns, and 4) assess the existing coverage in of these compounds in mass spectral database.

**Results:** We found that flame retardant classes as currently defined by the National Academies of Science, Engineering and Medicine (NASEM) are structurally diverse, with highly variable predicted pharmacokinetic properties and metabolic fates among member compounds. The vast majority of flame retardants (96%) and their predicted metabolites (99%) are not present in spectral databases, posing a challenge for identifying these compounds in human biosamples. However, we also demonstrate the utility of publicly available *in silico* methods in generating a fit for purpose synthetic spectral library for flame retardants and their metabolites that have yet to be identified in human biosamples.

**Discussion:** In conclusion, exposomics studies making use of fit-for-purpose synthetic spectral databases will better resolve internal exposure and windows of vulnerability associated with complex exposures to flame retardant chemicals and perturbed neurodevelopmental, reproductive, and other associated apical human health impacts.

## Introduction

The human exposome has been described as the totality of environmental exposures across an individual’s lifespan. It encompasses external factors including chemical, microbial, and radiation exposures, as well as internal factors such as metabolism, microbiome, and nutritional status that may modulate the effects of xenobiotic stressors on human health (Sillé et al., 2020). In practice, the positive identification of xenobiotics and their metabolites in human biosamples, a core element of exposomics, is complicated by the sheer quantity and diversity of measured compounds, which are often present at trace concentrations, as well as the absence of a wholly comprehensive database of chemical structures and properties to aid in the annotation of suspect compounds with spectral techniques.

The requisite experimental spectra–spanning multiple collision energies, polarity modes, and spectrometry platforms–for adequate compound identification simply does not exist for the universe of 160 million known chemicals and their human metabolites^45^. However, several databases enable identification of unknown compounds in untargeted mass spectrometry experiments from their m/z values and retention time and/or ion annotation information. The most comprehensive publicly available repository of mass spectrometry data is the METLIN database maintained by the Scripps Research Institute. The library contains 960,000 compounds annotated with chemical and molecular formulas, structural identifiers and high-resolution tandem mass spectral data produced in positive and negative ionization modes at different collision energies, collectively comprising over 4 million individual mass spectra (Xue et al., 2020; Scripps Research Institute, 2023). While substantial, METLIN chemical coverage represents less than a 1% coverage of the known chemical universe according to PubChem and consists mainly of chemicals with low relevance to environmental health (U.S. National Library of Medicine, 2023; Wang et al., 2022). Other databases offer substantially narrower chemical coverage. The Human Metabolome Database (HMDB) contains chemical, clinical, molecular, and spectral data for roughly 220,000 known metabolites, and allows users to both obtain *a priori* predicted or empirical GC-MS, MS/MS, NMR, and IR spectral information for target compounds, and to input their own peak lists of unidentified compounds to compare against the HMDB library (Wishart et al., 2022). The NIST 23 electron ionization, MS/MS and gas chromatography libraries cover as many as 347,100 unique compounds (National Institutes of Standards and Technology, 2020). Where compounds can be identified in biosamples, exposure databases such as the Blood Exposome Database (BEDB) and the International Agency for Research on Cancer’s (IARC) Exposome Explorer database provide indications of internal exposure over time (International Agency for Research on Cancer IARC and University of Alberta, 2012).

Despite open access to these important resources, it is typical for fewer than 2% of spectra to be successfully annotated in an untargeted metabolomics investigation (da Silva et al., 2015). In all cases, existing resources are biased away from compounds that have yet to be substantially reported in the scientific literature, creating a chicken and egg scenario for the novel identification of xenobiotic exposure: if a compound is understudied it is functionally invisible in untargeted analyses, and it will remain understudied because its absence as a variable hinders the identification of a potential adverse biological effect.

This challenge is particularly apparent in the context of adequate hazard assessment of flame retardants. Flame retardants are a diverse category of chemicals that alter the normal degradation or combustion of flammable materials to reduce or eliminate their tendency to ignite when exposed to heat or flame (Organisation for Economic Co-operation and Development OECD, 2017). They are commonly added to furniture, carpeting, textiles, vehicle interiors, electronics, car seats, mattresses, and other consumer products, and often in high amounts (National Academies, 2019). Flame retardants comprise 4%–5% of furniture foam by weight, and flame retardants in plastic TV casings have been measured at 10%–15% by weight (Dishaw et al., 2014).

Flame retardants are united by common functionality rather than by a shared chemical structure or molecular composition, owing to the need to mitigate fire hazards posed by synthetic polymers with distinct chemical and physical properties and operating under dissimilar use scenarios (National Academies, 2019). In practical application, commercial flame retardant formulations use combinations of halogenated and nonhalogenated compounds, organophosphates, nitrogen-based compounds, inorganics and organics within the same product to leverage synergistic effects and combine different mechanisms of action. Most populations, therefore, are exposed to these chemicals as complex mixtures (European Chemicals Agncy ECHA, 2023; Pinfa, 2023).

Biomonitoring studies over the past decade indicate widespread internal exposure in American adults and children, with roughly a dozen diverse organophosphate and polybrominated compounds consistently present in 70%–100% of biofluids (Ospina et al., 2018; Wang et al., 2019; Eick et al., 2021). Such persistent measurements are likely a consequence of both flame retardants’ ubiquity, and their tendency to be physically applied to target materials rather than reactively integrated into a product’s polymeric backbone to create inherently flame-resistant materials. Exposure then results from diet, inhalation and exposure to dust. Because of their size, hand-to-mouth behavior, and time spent indoors, children often have a higher body burden than adults (Lunder et al., 2010; Stapleton et al., 2014).

Because of the complexity of exposure scenarios, the diversity of chemicals involved, and the fact that most people are exposed to multiple flame retardants simultaneously, establishing a clear link between exposures and outcomes has been challenging. Nonetheless, human epidemiological studies have implicated flame retardants as (neuro-)developmental and reproductive toxicants, endocrine disruptors, and carcinogens, with impaired outcomes stably observed across chemical subtypes and independent of whether the parent compound or its presumptive metabolite are measured. A 2020 review of human epidemiological literature concluded that pre- and postnatal exposure to polybrominated diphenyl ethers (PBDE) and their metabolites negatively impact childhood intelligence, with prenatal exposures being additionally associated with inattention, poorer cognition, and externalizing behaviors (aggression, bullying, defiance, etc.) (Vuong et al., 2018). A 2019 study indicated that higher concentrations of urinary organophosphate ester flame retardant metabolites during pregnancy were associated with impaired fine motor and early language skills in early childhood (Doherty et al., 2019), while another found additional associations with reduced IQ and working memory, with the strongest associations generated from models that considered the molar sum of flame retardant’s metabolites (Castorina et al., 2017). Studies have also linked heightened exposure to various subclasses of organophosphate flame retardants and their metabolites to reduced maternal and neonatal thyroid hormone levels (Percy et al., 2021). With higher urinary organophosphate flame retardant metabolite concentrations, women undergoing *in vitro* fertilization are also substantially less likely to experience successful fertilization, implantation, clinical pregnancy, and live birth (Carignan et al., 2017).

However, no studies to date have comprehensively measured exposure to all flame retardants or their breakdown products. Our already limited understanding of the hazards of these compounds are constrained to investigations into roughly two dozen OFR and BDE flame retardants and their major metabolites–a small subset of the 746 unique compounds and mixtures identified as flame retardants under the joint US Consumer Product Safety Commission (CPSC)-US Environmental Protection Agency (EPA) Flame Retardant Inventory^22^. The majority of flame retardants have not been investigated for their presence in human biosamples, do not have a robustly characterized toxicity profile in human, *in vitro* animal, or *in vivo* studies, and do not have publicly available structural information due to proprietary protections. In considering the human exposome, the high correlation of flame retardants uniformly present in human biosamples and their tendency to metabolize into the same metabolites, present two particularly relevant challenges in accurately identifying potential adverse health outcomes associated with flame retardant exposure (Wang et al., 2019). Without an *a priori* appraisal of which compounds to look for, targeted assessment of the flame retardant and their metabolic products cannot be adequately measured.

What is clear is that associations with adverse human health outcomes persist when measuring either parent flame retardant chemicals or their breakdown products, indicating a potential etiological role for either state. For instance, in a preliminary structural analysis of brominated flame retardants by the Danish EPA, the OECD (Q)SAR Toolbox identified structural alerts for carcinogenicity with a mutagenic or genotoxic mode of action for 61 structurally related compounds and many of their metabolites, indicating a common possible mechanism of action between parents and metabolites (Ministry of Environment and Food of Denmark, 2016). It is also known that some flame retardants are broken down to products that are themselves flame retardants, as is the case with diphenyl phosphate (DPHP) and halogenated compounds that undergo dehalogenation to lighter congerners (Wang et al., 2020).

Owing to concern about human health hazards from flame retardants and the challenges posed by the sheer number of different compounds that fall under this classification, in 2019 the NASEM produced a scoping report to identify known OFRs in commerce, and analyze whether OFRs can be treated as a single class (National Academies, 2019). The analysis concluded that OFRs cannot be distinguished as a single class as they do not have a common chemical structure or predicted biologic activity. Instead, it was suggested to proceed with 14 different subclasses, based in part on common chemical structure and presumed common bioactivity. However, as the report noted, there was some ambiguity in the class assignment, and in many instances lack of data prevented them from making strong conclusions about common biological activity.

Several computational tools exist to predict the phase I and phase II metabolism products of small organic compounds. BioTransformer is a publicly available command-line executable tool that facilitates metabolism prediction and metabolite identification for organic xenobiotics (Wishart et al., 2022). The software’s metabolite prediction tool utilizes a machine learning-enhanced rule-based approach to predict human phase I and phase II metabolism, promiscuous enzymatic metabolism, gut microbial metabolism, and abiotic environmental metabolism from reaction descriptions, rules, and constraints encoded as SMARTS and SMIRKS strings. Generalized chemical classification guides the selection of likely biotransformations, while physicochemical properties and structural fingerprints guide the prediction of substrate specificity. BioTransformer was trained using the MetXBioDB database of experimentally confirmed metabolites of drugs, pesticides, toxicants, and phytochemicals, and their concomitant biotransformations and biodegradations. While BioTransformer tends to overpredict the number of human metabolites generated as compared to other models available at the time of its publication (Meteor Nexus and ADMET Predictor), leading to a high false positive rate, the tool achieves far greater sensitivity (88%–90%) compared to the other models (13%–71%) (Djoumbou-Feunang et al., 2019). GLORYx is a web-based tool that operates in two parts: first, a machine learning based prediction model uses extremely randomized trees classifiers and 2-dimensional circular descriptors to generate site of metabolism predictions for phase 1 and phase 2 reactions (de Bruyn Kops et al., 2021). Then, transformation progressed according to a SMIRKS-based reaction rule set. GLORYx reaction rules were trained on the DrugBank and MetXBioDB databases, as well as tested on a curated dataset consisting of 37 pharmaceuticals. GLORYx has the added benefit of including metabolite priority scores based on the likelihood of generating any particular metabolite. Both BioTransformer and GLORYx accept structural input in SDF or SMILES format, and output predicted metabolites with identifiers, including InChi string, InChiKey, and canonical SMILES. As an additional benefit, GLORYx also provides metabolite prediction scores for the likelihood of generating each metabolite.

Computationally predicted spectra can aid in overcoming limitations imposed by poor database coverage of the environmental chemical space. For instance, the Competitive Fragmentation Modeling for Metabolite Identification (CFM-ID) web server enables the spectral prediction, compound identification, and peak assignment of target molecules with known structures (Wang et al., 2022). Specifically, CFM-ID 4.0 accepts SMILES or InChI string input and predicts fragmentation patterns and electrospray ionization quadrupole time-of-flight tandem mass spectra (ESI-QTOF-MS/MS) in either positive or negative ion mode, across four different collision energies (Wang et al., 2022). The server can also annotate target compounds in user-submitted mass spectral datasets.

Under ideal conditions, a read-across performed for risk assessment of functionally related compounds will be based on the molecular features that enable the compounds to have a specific mode of action. In the absence of a known mode of action for neurodevelopmental harm, US regulatory authorities are pursuing subclass-based hazard assessment of organohalogen flame retardants based on a read-across of compounds subclustered by shared structural features and limited ToxCast assay activity (National Academies, 2019). However, this class-based assessment does not take into consideration the role of potentially toxic metabolites, nor to expanding the identification of parent flame retardants and their metabolic breakdown products in human biosamples to better inform the biology of neurodevelopmental hazards imposed by these compounds. It is further uncertain whether proposed organohalogen subclass definitions also sufficiently separate metabolites, or whether metabolites can originate from several classes -- including nonhalogenated compounds not covered by the current regulatory strategy. There are concerns that regulating compounds as a group, if not clustered on the basis of known, shared mechanism of toxicity, risks missing potentially bioactive parent compounds or their metabolites, while simultaneously restricting access to biologically neutral compounds in a way that incentivizes use of compounds with overlooked toxicity.

Here, we show that *in silico* methods commonly used for novel compound identification can play a useful role in identifying flame retardants and their metabolites that have yet to be identified in human biosamples. By using publicly available *in silico* tools, we were able to predict the metabolic breakdown products of discrete organic flame retardant compounds, identify whether these metabolites were shared across the parent subclasses as put forth by the National Academies of Science report, assess the existing coverage of these compounds in mass spectral databases, and compare the empirical and predicted spectral information of parent flame retardants and predicted metabolite compounds for use in untargeted exposomics studies (National Academies, 2019). We also show that the class-based approach is challenging both in terms of defining chemical moieties, predicted pharmacokinetics, and likely metabolic fate, and that while some parent compounds shared common metabolites there was an enormous diversity of potential metabolites. More broadly speaking, the sheer quantity and diversity of metabolites present a challenge analytically, and existing databases have fairly minimal coverage of potential flame retardant metabolites. We suggest demonstrate that *a priori* experimental integration of publicly available *in silico* methods for metabolite prediction can streamline one of the significant bottle-necks of exposomics studies by improving accuracy of compound identification in in human biosamples.

## Methods

### Parent flame retardant chemical space

The inventory of 746 likely flame retardants was obtained from the Joint US Consumer Product Safety Commission (CPSC)-US Environmental Protection Agency (EPA) Flame Retardant Inventory, available through the EPA CompTox Chemicals Dashboard and described in detail in Bevington et al., 2022 (United States Environmental Protection Agency U.S. EPA, 2023; Bevington et al., 2022). Filters provided in the Bevington publication were used to annotate the CompTox inventory list to remove inorganic compounds and mixtures from the inventory, leaving 601 organic flame retardants.

A QSAR-ready SMILES string is a standardized format for representing desalted, stereo-neutral molecules for use in cheminformatics. For inventory compounds exported from CompTox with no or a corrupted QSAR-ready SMILES string, the Bevington publication was consulted and QSAR-ready SMILES strings were obtained for an additional 24 compounds (Bevington et al., 2022). The webchem R package was used to obtain canonical SMILES from the NIH Chemical Identifier Resolver (CIR) through matching CASRN or INCHIKEY for the remaining compounds with missing QSAR-ready SMILES, and the rcdk R package was used to convert canonical SMILES to SDF for use in the KNIME Standardization workflow for QSAR-ready chemical structures pretreatment. 38 new structures were resolved, permitting a total of 550 compounds to be assessed for bioavailability and biotransformation. Of these compounds, 525 were unique.

Parent flame retardants were assigned to one of fifteen classes based on the strategy of shared ToxPrint chemotypes and ToxCast biological activity outlined by the National Academies of Sciences, Engineering, and Medicine (NASEM) in the 2019 report, “A Class Approach to Hazard Assessment of Organohalogen Flame Retardants” (National Academies, 2019). While this report—and CPSC’s subsequent regulatory strategy—focus primarily on subclassifying organohalogen flame retardants, our analysis includes organic nonhalogenated compounds assigned to a single nonhalogenated class. Since the scope of this paper extends beyond organohalogenated flame retardants, we will refer to all chemical groupings as “classes,” rather than “subclasses,” as they are designated in the NASEM report.

In the course of generating QSAR-ready SIMLES strings, 35 flame retardants with a unique DTXSID were collapsed into 8 compounds. In a single case, two flame retardant compounds from different NASEM classes were collapsed into a single QSAR-ready structure (DTXSID201016707, a member of the polyhalogenated benzene aliphatics and functionalized class, and DTXSID501016706, a member of the polyhalogenated carbocycles class). Such a scenario was not unexpected, because it was noted by NASEM in their initial exploration of organohalogen flame retardant classification that some compounds meet the definition of multiple classes (National Academies, 2019). The compound was assigned to the polyhalogenated benzene aliphatics and functionalized class for purpose of this analysis, because it shares a larger common organic backbone with other members of this class than the polyhalogenated carbocycles.

### Tanimoto similarity

The RDKit chemoinformatics package (v.2022.09.5) was used to calculate RDKIT-type of fingerprints (maxPath = 5, fpSize = 2048). Subsequently, the fingerprints were used to obtain pairwise Tanimoto similarity matrix. Metabolite Predictions.

The open-access BioTransformer 3.1.0 software and GLORYx tool were used to facilitate *in silico* metabolism prediction and identification of organic flame retardants (Stork et al., 2019; de Bruyn Kops et al., 2021; Wishart et al., 2022). For BioTransformer, metabolites were predicted from parent QSAR-ready SMILES strings using the metabolic transformation parameter AllHuman in combined Cyp450 mode with single step iteration. This effectively predicts biotransformation products of CYP450s, promiscuous enzymes, phase II, and gut microbial metabolism in a single step. Predictions originating from gut microbial metabolism were not considered in the scope of this publication. For GLORYx, the products of a single iteration of phase I or phase II metabolism were predicted. The GLORYx output can be found online at https://nerdd.univie.ac.at/gloryx/result/6082ad54-a1a7-438a-942c-f60f1c5fe139. Parent OFR and metabolite InChI Keys were used sequentially to match metabolites identified across both models.

### Parent flame retardant and metabolite network analysis

Parent-metabolite networks and maximum common substructure (MCS) of NASEM-defined parent flame retardant classes were generated from QSAR-ready SMILES strings using Cytoscape 3.9.1 and the ChemViz accessory application (Shannon et al., 2003; Morris and Jia, 2023).

### Pharmacokinetic predictions

The SwissADME online tool was used to predict gastrointestinal (GI) absorption and blood brain barrier (BBB) permeability for flame retardants and their metabolites, using QSAR-ready SMILES strings as the structural input (Daina et al., 2017).

### Database coverage

The Chemical Translation Service (CTS) was used to convert parent flame retardant and metabolite identifiers (CASRN and InChiKey where available) to Human Metabolome Database (HMDB) IDs (Wohlgemuth et al., 2010). All hits were manually quality checked in HMDB.

### Spectral prediction

The CFM-ID 4.0 server was used to generate synthetic spectra and m/z values for electrospray ionization (ESI) and used as a comparator for empirical LC-MS/MS data (Wang et al., 2022). Where possible, synthetic spectra were produced under the same experimental conditions (spectrum type, ion mode, collision energy) enumerated in empirical spectra deposited in HMDB.

## Results

### Parent flame retardant chemical space

**FIGURE 1:**
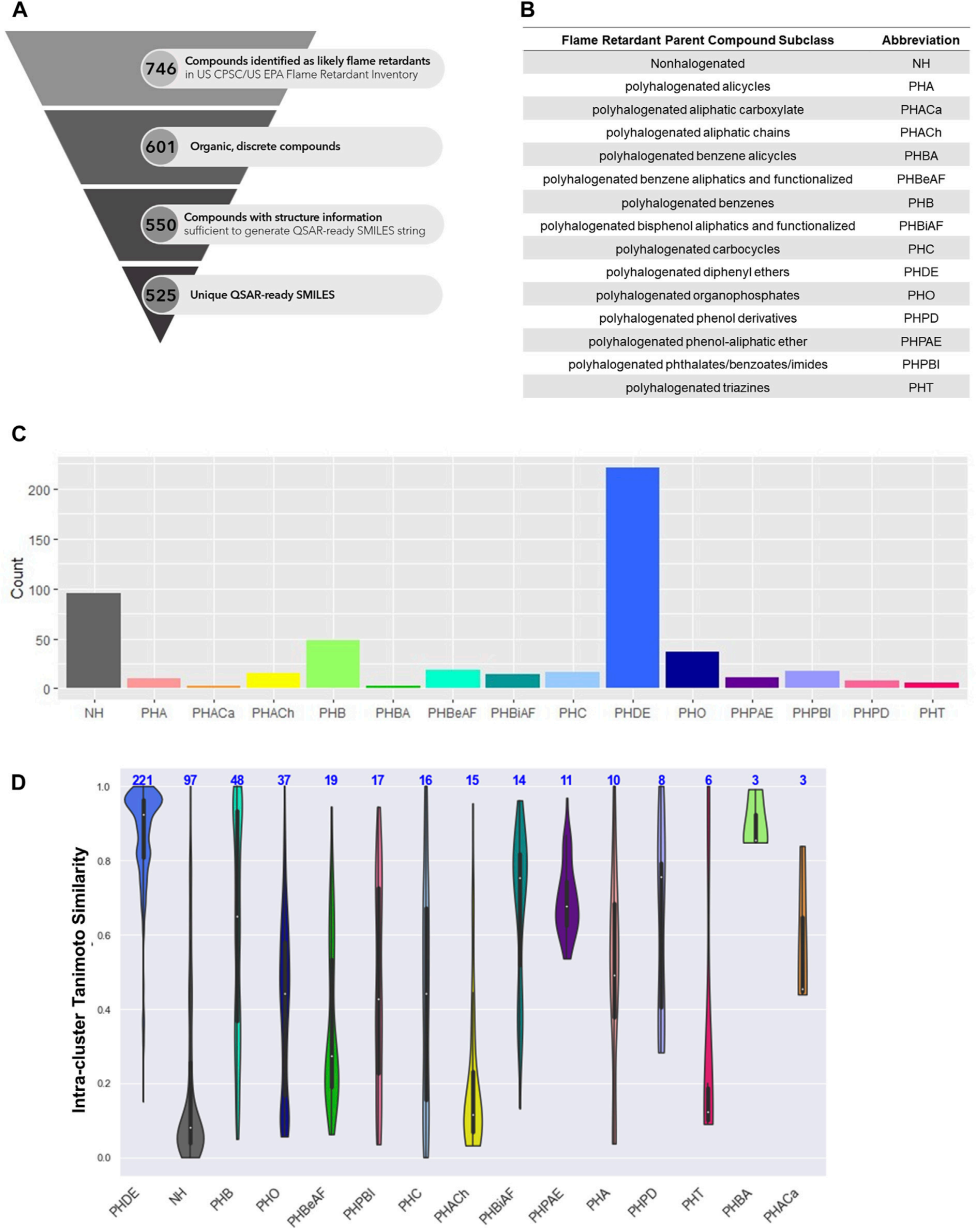
**(A)** Discrete organic flame retardant chemical space. **(B)** Abbreviation of National Academies of Science Engineering and Medicine (NASEM) defined classes. **(C)** Number of flame retardant chemicals by NASEM-defined class. **(D)** Intra-class Tanimoto similarity. Overall, 525 chemicals were used in this analysis, and **(A)** summarizes the procedure to prune the initial data set of 746 chemicals from the US CPSC/US EPA Flame Retardant inventory down to the final data set of 525 unique chemicals QSAR-ready SMILES. **(B)** presents the abbreviations of National Academies of Science Engineering and Medicine (NASEM) defined class names. **(C)** presents the distribution of the number of chemicals by class according to NASEM, with the majority of the chemicals belonging to the polyhalogenated diphenyl ethers class. The structural similarity within each class is displayed in **(D)**. Despite chemicals belonging to the same chemical class, there is quite a range of structural similarity (using Tanimoto distance) within each class (e.g., 0%–100% in the polyhalogenated carbocycles), with the exception of the polyhalogenated benzene alicycles, which were 85% or greater structurally similar to one another.

The Joint US Consumer Product Safety Commission (CPSC)-US Environmental Protection Agency (EPA) Flame Retardant Inventory contains 746 likely flame retardants, 601 of which are discrete, organic compounds. All 601 compounds were identifiable by EPA’s DSSTox substance identifier (DTXSID), 599 were identifiable by CAS registry number (CASRN), 533 by SMILES string, and 513 by QSAR-ready SMILES string. Of these flame retardants, identifier matching and the KNIME standardization workflow was successful in resolving QSAR-ready SMILES strings for 550 compounds, 525 of which were unique (Figure 1A). Compounds for which a QSAR-ready SMILES string could not be resolved were either mixtures such as Firemaster 550 (DTXSID70880073) and Antiblaze 19 (DTXSID80107622), inorganic compounds such as Titanium tetrachloride (DTXSID8042476) and Ammonium phosphate (DTXSID5029689), or poorly defined compounds with highly variable structures, such as Chloroalkanes C10-​14 (DTXSID70872670) and Hexabromobiphenyl (DTXSID3025382), for which no SDF structure was available.

All parent compounds were assigned to a single class for the purpose of this analysis (Figure 1C). Polyhalogenated diphenyl ethers were overrepresented with 221 member compounds, comprising 42% of the chemical space. Nonhalogenated compounds were the next most abundant at 97 (18%), followed by polyhalogenated benzenes at 48 (9%), and polyhalogenated organophosphates at 37 (7%). The remaining eleven classes (polyhalogenated aliphatic carboxylates, polyhalogenated benzene alicycles, polyhalogenated triazines, polyhalogenated phenol derivatives, polyhalogenated alicycles, polyhalogenated phenol-aliphatic ether, polyhalogenated bisphenol aliphatics and functionalized, polyhalogenated aliphatic chains, polyhalogenated carbocycles, polyhalogenated phthalates/benzoates/imides, polyhalogenated benzene aliphatics and functionalized) contained 19 or fewer compounds and each represented less than 5% of the parent flame retardant chemical space.

**FIGURE 2:**
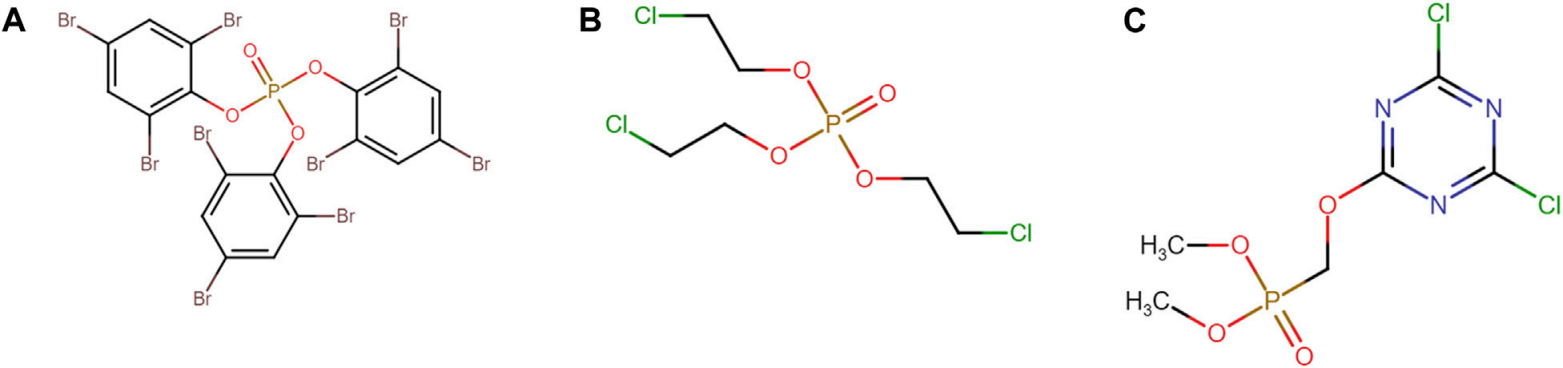
Chemical structure of three members of the polyhalogenated organophosphates class. **(A)** Tris (2,4,6-tribromophenyl) phosphate, **(B)** Tris (2-chloroethyl) phosphate and **(C)** Dimethyl (4,6-dichloro-1,3,5-triazin-2-yloxy)methylphosphonate are all classified under the NASEM strategy as polyhalogenated organophosphates despite containing either aliphatic or aromatic substituents.

Intra-class Tanimoto similarity indicates that the polyhalogenated diphenyl ethers form the largest and most homogenous group of flame retardants (mean = 0.87, N = 221) (Figure 1D). Though smaller, the polyhalogenated bisphenol aliphatics and functionalized (mean = 0.68, N = 14) and polyhalogenated benzene (mean = 0.62, N = 48) classes are also quite homogenous. Unsurprisingly, the large nonhalogenated class is very heterogeneous. It should be noted that the structural homogeneity varied between flame retardant classes. For instance, the group of polyhalogenated organophosphates had both aliphatic and various aromatic substituents (Figure 2).

These structural differences within a group, despite sharing common substructure, may lead to significant differences in xenobiotic metabolism. This may result in the presence of metabolites with relatively large differences in ADMET profiles. Considering this, it could be argued that some of the original clusters could be further subclassified.

Our analysis of the MCS of each parent flame retardant class showed the challenge of making consistent distinctions amongst a relatively diverse group of organic chemicals. While for some of the smaller and less diverse classes (for example, benzene alicycles) the MCS did identify the common organic backbone that would be considered the defining feature of the class (Supplementary Figure S1). However, for the larger and more diverse classes the MCS was minimal–for example, for the polyhalogenated aliphatics and functionalized class the common backbone was benzene, and for the polyhalogenated organophosphate class it was only oxygen as the organophosphate structure were differently substituted. Moreover, while the presence of an organophosphate moiety might seem like a reasonable criterion for a class membership, two of the organophosphates were aromatics and structurally would be equally well placed in a category with more aromatics.

### Metabolism predictions

**FIGURE 3:**
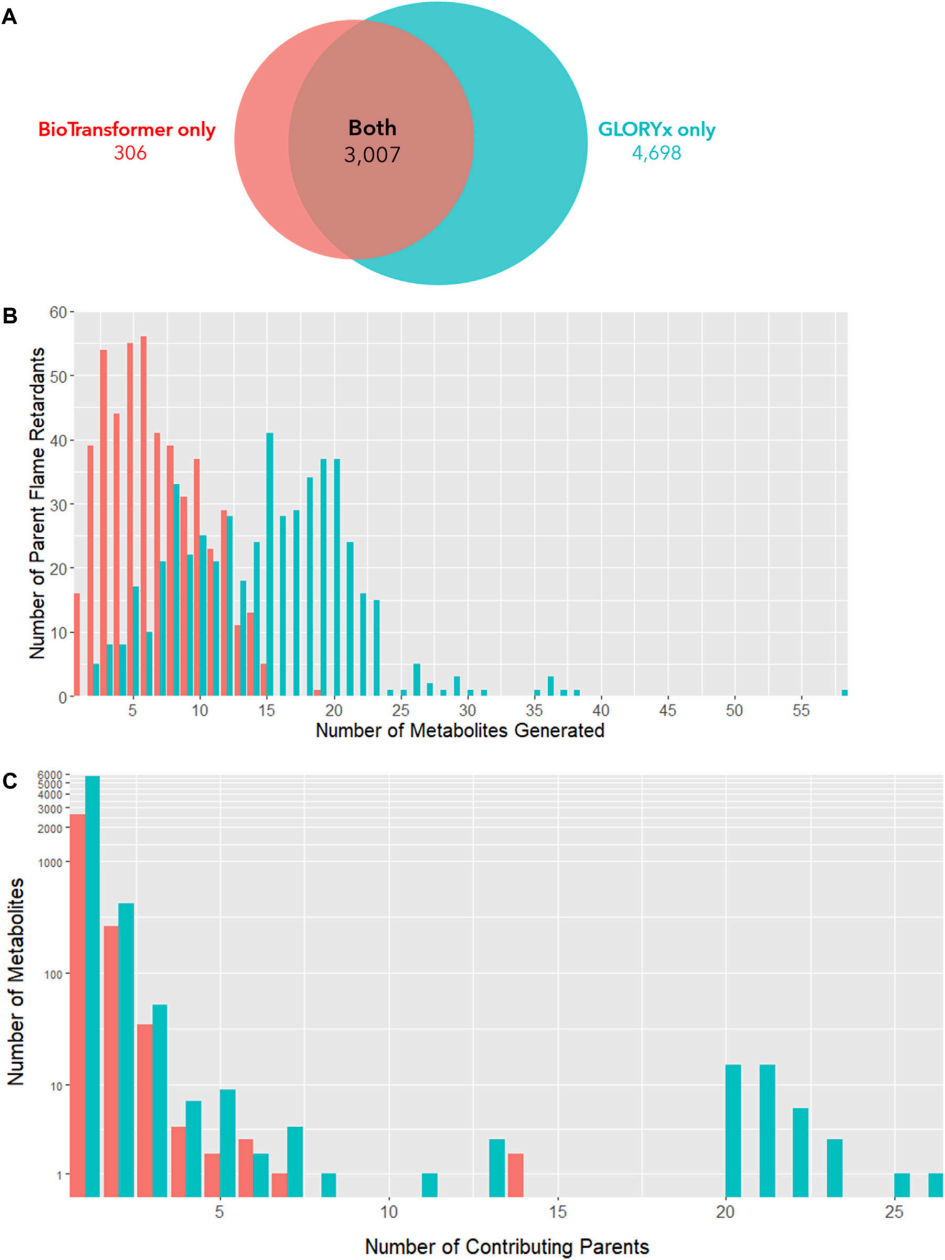
**(A)** Metabolite-parent predictions from BioTransformer and GLORYx. **(B)** Number of metabolites produced by each parent flame retardant. **(C)** Number of individual parent flame retardants contributing to each metabolite. **(A)** presents the overlap of the metabolites produced by the two *in silico* metabolism simulators, BioTransformer and GLORYx. GLORYx predicted far more metabolites than BioTransfomer (N = 3,313 vs. N = 7,705). Nearly all metabolites predicted to form according to BioTransformer were also predicted by GLORYx (N = 3,007 overlap). **(B)** shows the distribution of parent flame retardant compounds that generate a given number of metabolites. GLORYx (teal) on average predicts the formation of more metabolites per compound than BioTransformer (red) due to its inclusion of lower probability products (μ = 14.7 vs. 6.7 metabolites per parent flame retardant). **(C)** depicts the number of individual parent flame retardants that generate each unique metabolite. Under both models, the vast majority of metabolites originate from a single parent compound, or from a limited number of parent compounds.

After removing metabolites originating only from gut microbial metabolism, BioTransformer was able to identify metabolites for 494 parent compounds. 3,311 individual reactions resulted in 2,912 unique metabolites identified in total. 309 metabolites originated from multiple parent OFRs, while 2,603 metabolites originated from a single parent OFR. Comparatively, GLORYx was able to positively identify metabolites for 523 parent compounds. 7,704 individual reactions resulted in 6,213 unique metabolites identified in total. 537 metabolites originated from multiple parent OFRs, while 5,676 metabolites originated from a single parent OFR. 3,007 parent-metabolite pairs were predicted in both models, while BioTransformer alone identified an additional 304 pairs and GLOXYx alone identified 4,697 pairs. In total, 6,474 unique metabolites were predicted between both models (Figure 3A). GLORYx predicted 14.7 metabolites generated per flame retardant compared to 6.7 by BioTransformer (Figure 3B). The discrepancy between models can likely be explained by GLORYx’s inclusion of more low probability biotransformation products in its predictions. While all metabolites were considered in this analysis, it is important to note that among the products predicted by GLORYx as the most likely metabolites to be generated (i.e., those with a priority score above 0.5), over 90% of were also predicted by BioTransformer, indicating a high concordance among the most likely candidate compounds.

Under both models, the majority of metabolites originate from a single parent compound (Figure 3C). However, 309 BioTransformer metabolites and 537 GLORYx metabolites were predicted to form from the metabolism of more than one flame retardant parent compound. This suggests that coexposure to multiple flame retardants, as occurs in contemporary exposure scenarios, may result in exposure to the same biotransformation product. For the more extreme scenario presented by 6 of these metabolites, more than 20 parent compounds can potentially contribute to their generation.

**FIGURE 4:**
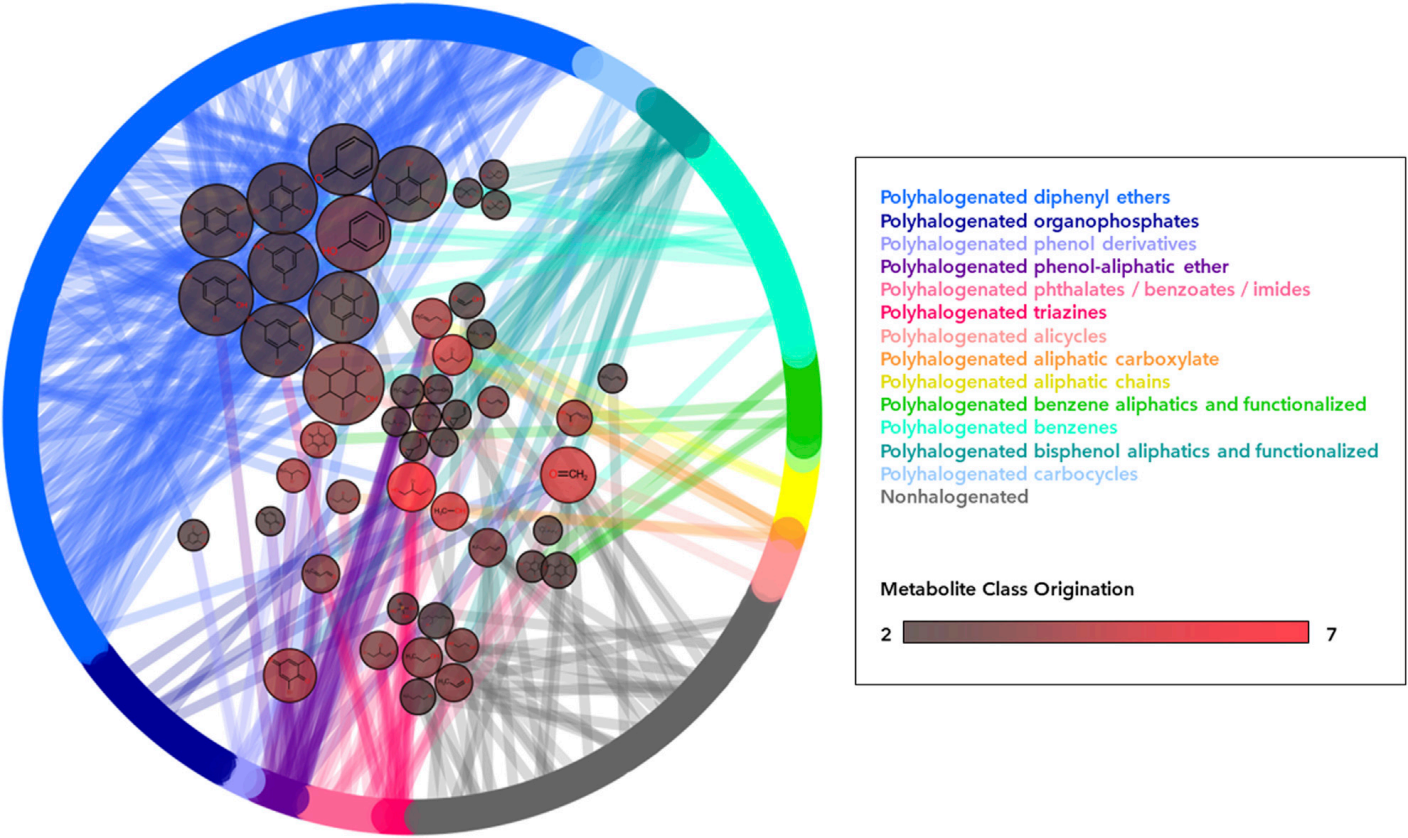
Network of common metabolite generation across flame retardant classes. All parent flame retardant compounds (525) are arranged as nodes in the outer ring and colored according to their corresponding class. Edges connect flame retardant parents to their metabolite product, arranged as nodes in the center pool. Metabolite node color indicates the number of different parent compound classes (1–7) capable of generating this specific metabolite, with brighter red nodes indicating more classes contributing. Metabolite node size indicates the total number of flame retardant parent compounds (1–26) capable of generating this specific metabolite. Only metabolites that are the product of two or more distinct parent classes are pictured in order to demonstrate cross-class production of metabolites.

In terms of metabolite commonality between disparate flame retardant sublass designations, the vast majority of metabolite products (6,429; 99%) were unique to a single flame retardant class, while 48 products (1%) were generated from parent compounds in different classes (Table 1). Where class overlap exists in metabolite generation, flame retardants from the same two or three classes often produce several shared metabolite products among them (Figure 4). For instance, the polyhalogenated bisphenol aliphatics and functionalized class shares 14 metabolites with the polyhalogenated phenol-aliphatic ethers and 8 with the polyhalogenated diphenyl ethers. Nonhalogenated compounds also shared 13 metabolites with members of 9 polyhalogenated classes. While most of these are low molecular weight compounds (i.e., Formaldehyde, carbon monoxide), some such as phosphoric acid, 2-ethylhexan-1-ol, acetaldehyde, and phenol may be of toxicological relevance.

**TABLE 1:** Metabolites classified according to parent compound class. Most predicted metabolites (N = 6,429) were unique to a single compound class, while a minority (N = 48) were capable of being generated from parent compounds in multiple classes.

Unique classes	Number of metabolites	%
**1**	6,429	*99.25*
**2**	29	*0.44*
**3**	10	*0.15*
**4**	5	*0.07*
**5**	3	*0.04*
**6**	0	*0*
**7**	1	*0.02*

The italic values as indicated the % is percent of total metabolites generated.

In rare cases, single metabolites are generated from numerous classes. Model predictions indicate that 11 flame retardants from 7 distinct parent classes were capable of generating 2,3-Dibromo-1-propanol (2,3-DBP, DTXSID7021817), which the US National Toxicology Program (NTP) classifies as reasonably anticipated to be a human carcinogen (National Toxicology Program NTP, 2021). 2,3-DBP is also a known human metabolite of the flame retardant tris(2,3-dibromopropyl) phosphate, but is not present in HMDB and has not yet been empirically associated with the remaining 10 generating parent flame retardants either in human biosamples or *in vitro* assessments.

**FIGURE 5:**
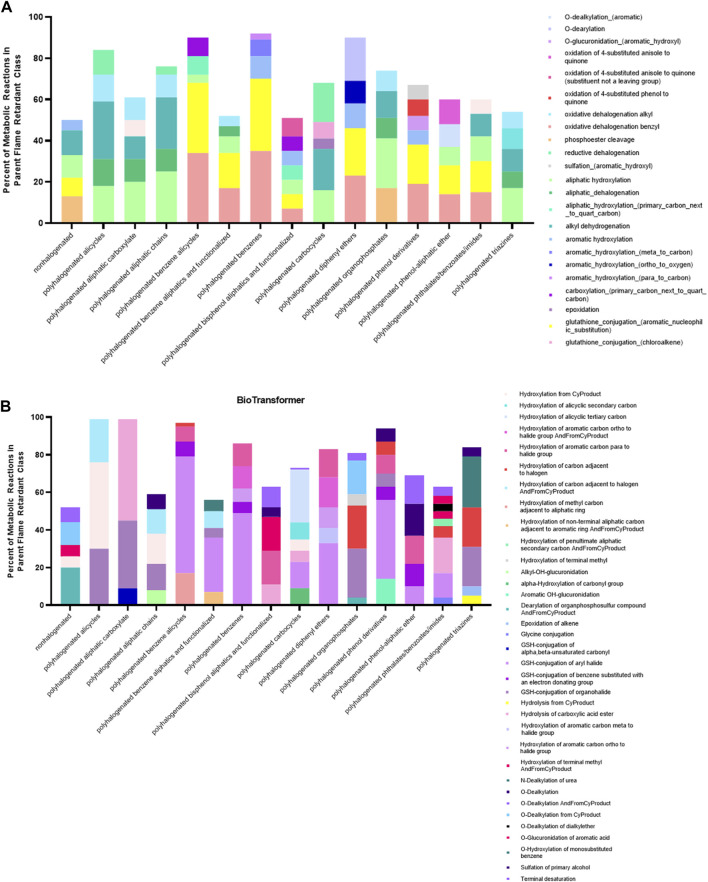
Prominent reaction types in BioTransformer **(A)** and GLORYx **(B)** predicted metabolism. Despite structural similarity, flame retardant class members were subject to a diversity of reaction types. The top 5 most common reaction types (6 where there was a tie) predicted by each model cover only 50%–80% of all reactions within most classes. GLORYx predicted an outsized number of phase II reactions compared to BioTransformer.

While we initially anticipated a relatively consistent set of reaction types within each parent class due to some degree of shared molecular structure, both GLORYx and BioTransformer predicted parent flame retardants to be subject to an extremely diverse set of metabolic reactions. The most commonly occurring reaction types within each class are presented in Figure 5. Both models predicted a relatively high number of phase II reactions resulting in the direct conjugation of parent flame retardants to highly polar antioxidants, with 1,595 phase II reactions predicted by GLORYx and 1,137 predicted by BioTransformer. In terms of reaction types common to those metabolites that are generated from multiple different classes, the most prominent across both models were O-dearylation, O-dealkylation (both aliphatic and aromatic), oxidative cleavage to an alcohol and one aldehyde or ketone, oxidation to a quinone, phosphoester cleavage, and hydrolysis of esters and other less common functional groups.

Interestingly, 7 flame retardants were predicted to be metabolized–typically via dealkylation, phosphoester cleavage, or hydrolysis–into structures that were other flame retardants. This would significantly complicate measuring exposure as it would be difficult to isolate exposures vs. metabolites; moreover, exposure could be potentially underestimated if, for example, only one parent compound was analyzed in a dust sample without appreciating that another flame retardant, after biotransformation, could result in a significantly elevated internal dose. This is particularly relevant for the flame retardant 2,3-DBP, which is itself a metabolite of 11 other compounds across 7 classes.

We validated our predicted metabolites against experimentally observed metabolites for two well-studied flame retardant compounds: 2.2′, 4.4′-Tetrabromodiphenyl ether (BDE-47) from the polyhalogenated diphenyl ether class, and 3.3′, 5.5′-Tetrabromobisphenol A (TBBPA) from the polyhalogenated bisphenol aliphatics and functionalized class. We first searched PubChem for PubMed literature indexed to each metabolite, then expanded our search to PubMed for non-indexed studies using search terms inclusive of proposed general reaction types (i.e., (“3.3′, 5.5′-Tetrabromobisphenol A” OR “TBBPA”) AND “glucuronidation”). For BDE-47, 8 phase I metabolites were predicted by BioTransformer and GLORYx. All but one metabolite were found in the literature, and of those found all 7 were of mammalian origin. No phase II metabolites were found by querying on structure, but an EPA toxicological summary of possible metabolic routes did mention potential glutathione conjugates (Supplementary Data). The models identified 9 purported metabolites of TBBPA, including the known primary human glucuronidation and sulfation products. In total, experimental evidence was found for 3 products of phase I metabolism and 4 products of phase II metabolism (Schauer et al., 2006; Zalko et al., 2006; Jaro et al., 2019). Evidence for two TBBPA metabolites, the quinone and methylation product, was only found in invertebrates and microbes. However, all other metabolites were evident in human studies (Supplementary Data). A review of the TBBPA metabolism literature also revealed several products of human metabolism obtained *in vitro*, from liver microsomes, or S9 fractions whose formation was not predicted due to formation requiring multiple phases of biochemical reactions, such as a glutathione conjugate of 2,6-dibromo-4-isopropyl phenol (Schauer et al., 2006; Zalko et al., 2006; Jaro et al., 2019; Smythe et al., 2022). These metabolites, while potentially toxicologically relevant, were beyond the scope of this investigation.

### Predicted pharmacokinetics

SwissADME is an online tool that aggregates the output of several leading *in silico* methods for physicochemical property and pharmacokinetic parameter prediction, including gastrointestinal (GI) absorption and blood brain barrier (BBB) permeability (Daina et al., 2017). Concordance across these two measures was low among most classes and did not improve in classes with high Tanimoto similarity (Table 2). Three of the smaller classes, polyhalogenated alicycles, polyhalogenated aliphatic carboxylates, and polyhalogenated benzene alicycles, exhibited complete concordance for predicted GI absorption and BBB permeability. Tanimoto similarity indicates that the polyhalogenated alicycles are highly homogenous, while the latter two classes exhibit only moderate structural similarity. The largest and most homogenous class, polyhalogenated diphenyl ethers, also exhibited high concordance among predicted GI absorption and BBB permeability, while the next most homogenous class, the polyhalogenated phenol-aliphatic ethers, were split 1:2 for GI absorption and almost 1:1 for BBB permeability.

**TABLE 2:** Predicted Pharmacokinetic Properties of Parent Flame Retardants. SwissADME-predicted gastrointestinal (GI) absorption and blood brain barrier (BBB) permeability of flame retardants by class reveal a diversity of expected bioavailability.

Class	Total compounds	GI absorption				BBB permeant			
		Low	*%*	High	*%*	No	*%*	Yes	*%*
nonhalogenated	97	33	*34%*	64	*66%*	68	*70%*	29	*30%*
polyhalogenated alicycles	10	10	*100%*	0	*0%*	10	*100%*	0	*0%*
polyhalogenated aliphatic carboxylate	3	0	*0%*	3	*100%*	0	*0%*	3	*100%*
polyhalogenated aliphatic chains	15	10	*67%*	5	*33%*	7	*47%*	8	*53%*
polyhalogenated benzene alicycles	3	3	*100%*	0	*0%*	3	*100%*	0	*0%*
polyhalogenated benzene aliphatics and functionalized	19	16	*84%*	3	*16%*	15	*79%*	4	*21%*
polyhalogenated benzenes	48	46	*96%*	2	*4%*	42	*88%*	6	*13%*
polyhalogenated bisphenol aliphatics and functionalized	14	8	*57%*	6	*43%*	13	*93%*	1	*7%*
polyhalogenated carbocycles	16	10	*63%*	6	*38%*	12	*75%*	4	*25%*
polyhalogenated diphenyl ethers	221	203	*92%*	18	*8%*	204	*92%*	17	*8%*
polyhalogenated organophosphates	37	10	*27%*	27	*73%*	16	*43%*	21	*57%*
polyhalogenated phenol derivatives	8	1	*13%*	7	*88%*	2	*25%*	6	*75%*
polyhalogenated phenol-aliphatic ether	11	4	*36%*	7	*64%*	6	*55%*	5	*45%*
polyhalogenated phthalates/benzoates/imides	17	7	*41%*	10	*59%*	9	*53%*	8	*47%*
polyhalogenated triazines	6	1	*17%*	5	*83%*	3	*50%*	3	*50%*

The italic values as indicated the % is percent of FRs within that FR class.

### Database coverage

CTS was used to convert flame retardant and metabolite identifiers (CAS and InChIKey) to HMDB identification number, and all hits were confirmed with manual quality checks. Only 21 parent flame retardants (4.0%) and 69 metabolites (1.1%) returned positive matches. Interestingly, for 14 parent flame retardant and 11 metabolite hits, HMDB entries were populated only with predicted spectra–almost exclusively using CFM tools–and lacked any empirical spectra. Only one halogenated flame retardant, 2,6-dibromophenol from the polyhalogenated phenol derivatives class, was among the compounds with empirical spectral information, although substantial computationally-derived spectral data were deposited for the remaining flame retardant hits. In terms of measured human exposure captured by the BEDB, 330 parent compounds (63%) and 211 metabolites (3%) have been measured in human biosamples, indicating that empirical spectral data may exist in the published literature for these additional compounds, outside the HMDB.

### Predicted spectra

**FIGURE 6:**
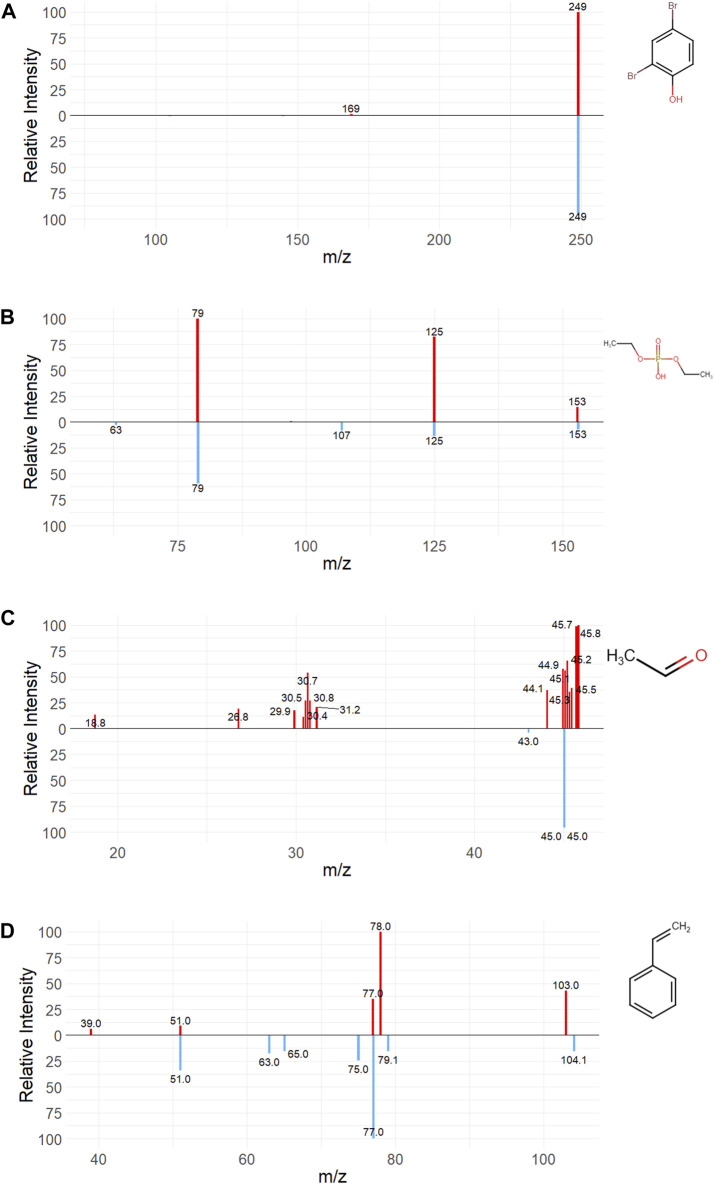
Comparison between predicted and empirical spectra. Empirical (red, top spectrum) and the most similar CFM-ID predicted spectrum (blue, bottom spectrum) for the flame retardants **(A)** 2,4-dibromophenol and **(B)** diethylphosphate, and the predicted metabolites **(C)** acetaldehyde and **(D)** styrene.

As an exercise in utilizing publicly available tools to predict spectral information, two flame retardants (2,4-dibromophenol and diethylphosphate) and two metabolites (acetaldehyde and styrene) with HMDB deposited experimental spectra were used to qualitatively assess the difference between empirical and computationally generated ESI spectra using the CFM-ID tool. Synthetic spectra were produced under the same experimental conditions (spectrum type, ion mode, closest collision energy voltage) enumerated in empirical spectra where possible. The experimental QTOF LC-MS/MS spectrum for 2,4-Dibromophenol was generated in negative ion mode at 35V, while the most similar synthetic spectra was based on a 10 V collision energy with a [M-H-] adduct (Figure 6A). The experimental LC-ESI-QQ spectrum for diethylphosphate was generated in negative ion mode with no available collision energy data, but matched most closely with the 20 V CFM-ID spectrum with a [M-H-] adduct (Figure 6B). The experimental QQQ spectrum for Acetaldehyde was generated in positive ion mode at 10 V collision energy, and the predicted spectrum was at the same collision energy voltage with a [M-H-] adduct (Figure 6C). The experimental LC-MS/MS spectrum for styrene was generated in positive ion mode at 50 V collision energy, while the predicted spectrum was generated at 40 V with a [M+] adduct (Figure 6D). CFM-ID performed fairly well identifying fragment m/z and peak intensity for parent flame retardant and styrene spectra, while it grossly underpredicted the number of fragments in the acetaldehyde spectra. This is likely due to the acetaldehyde spectrum originating from a QQQ. CFM-ID was trained on spectral data generated with a QTOF, and thus is more poorly predictive of Orbitrap or QQQ spectra.

## Conclusion

The goal of this paper was to use publicly available *in silico* tools to predict the metabolic breakdown products of discrete organic flame retardant compounds, to identify whether these metabolites were shared across the parent classes that will inform read-across for risk assessment, to assess the existing coverage in of these compounds in mass spectral databases, and to compare empirical and predicted spectral information of parent flame retardants and predicted metabolite compounds for general use.

Although the NASEM-guided halogenated flame retardant subclassing strategy was expert-derived and based on shared chemotypes and ToxCast biological activity where available, our analysis showed that among 525 discrete, organic flame retardants, the classes generally achieve poor Tanimoto similarity (i.e., below 0.85), share functionally nonspecific MCSs, have a diversity of biologically relevant substitution patterns, and exhibit poor concordance in predicted GI and BBB permeance.

First pass metabolite prediction with BioTransformer and GLORYx generated 6,474 unique metabolites, with model concordance above 90% when considering metabolites with a medium to high probability of forming. Experimental evidence for human or mammalian biotransformation of BDE-47 or TBBPA into their respective predicted metabolites was found for 14 of 17 compounds, constituting both phase I and phase II products. While this study only considered a single iteration of biochemical reactions, it is possible to generate transformation products from multiple rounds of metabolism to fully capture the universe of potential human metabolites. Such a strategy would, however, be computationally expensive due to the exponential increase in the number of compounds generated with each iteration.

Within classes, parent flame retardants were subject to an extremely diverse set of reaction types. Biotransformations yielding metabolites common to multiple classes tended to be those that catabolize a flame retardant into a large generic fragment, such as dearylation, dealkylation, and ester hydrolysis. A total of 48 unique metabolites were common across distinct classes, indicating that coexposure to multiple parent flame retardants, as would occur in everyday life, may result in excess exposure to common metabolites.

Clustering biologically similar compounds for the purpose of human health risk assessment is a scientifically valid and economically practicable strategy, but toxicological action must be at the forefront of cluster decision-making in order to meaningfully distinguish chemicals that may introduce an undue hazard under their current use scenarios from those which are benign. With limited biologically-relevant information available to inform flame retardant clustering strategies, this investigation provides evidence indicating that the frontrunning strategy for flame retardant classification may not sufficiently achieve this goal.

With coexposure scenarios in mind, the limited database coverage of parent flame retardant (4%) and metabolite (1%) spectral data presents a challenge for compound identification in untargeted exposomics studies. However, we qualitatively demonstrated the utility of using synthetic spectra as a stand-in when empirical data is not yet available for a compound of interest. This strategy of using predicted compounds to predict spectral information is an important novelty of this study, and may aid in the future novel identification of poorly studied chemicals and metabolites of toxicological interest in human biosamples. Such information is imperative to support ongoing efforts to identify environmental toxicants and their metabolites in human biosamples. Exposomics studies making use of such fit-for-purpose synthetic spectral databases will better resolve internal exposure and windows of vulnerability associated with complex mixtures of flame retardant chemicals, and perturbed neurodevelopmental, reproductive, and other associated apical human health impacts.

## Discussion

This study provides the first *in silico* prediction of metabolites for every flame retardant currently or formerly in commerce, as well as for compounds with flame retarding properties that may feasibly be used as flame retardants in the future. To our knowledge, our strategy of using computationally derived metabolites to generate synthetic spectra as a means of overcoming limitations in database coverage is also a novel strategy.

Flame retardants as a use category have been subject to extensive epidemiological study as well as *in vivo* and *in vitro* studies for mechanisms, yet there remains uncertainty about mechanisms, adverse outcomes, and benign levels of exposure. Some of the confusion no doubt stems from the limitation that most studies only consider partial exposures, while the nature of flame retardant mixtures present in a multitude of consumer items all but guarantees that individuals are exposed to many flame retardants simultaneously and ubiquitously. This is an important factor because not only do flame retardants share several common metabolites—including metabolites that are themselves flame retardants—but co-exposures have the potential to alter the rate of biotransformation by inducing enzymes or alternatively depleting glutathione.

One step forward for untargeted metabolomics or exposomics studies is to build databases that are fit for purpose, that capture potential xenobiotics as well as their purported metabolites and prioritizes compound identification based on likely exposures. While there is some progress towards this with databases such as the CompTox dashboard, there is as yet no comprehensive resource on the transformation products for chemicals in commerce. For many commercially important compounds, this data is simply lacking altogether.

Additionally, exposomics studies that rely on an untargeted approach to elucidate chemicals in human biomatrices depend on databases for compound identification, and if these databases lack the full catalog of possible metabolites, they will likely be unidentified. Computational tools that predict metabolism can therefore help to fill this gap. Tools like BioTransformer and GLORYx are best situated for casting a wide net of possible biotransformation products, to be later narrowed down based on in chemico, *in vitro*, or *in vivo* approaches for priority compounds. Here, we focused on first pass metabolism both to reduce the complexity of potential metabolites generated and because in many instances, breakdown products of first pass metabolism are likely to be more reactive. In addition, we excluded potential microbiome metabolites owing to a lack of information on the influence of the gut microbiome over xenobiotic metabolite load.

Flame retardant metabolite generation for toxicological purposes has not been extensively studied. A 2020 computational evaluation of the hazard ranking of six flame retardants and their metabolites found that every flame retardant was capable of being metabolized into a compound that was more hazardous than the parent (Zheng et al., 2021). In addition to human metabolism, the authors considered mammalian, microbial, and abiotic transformation using the Meteor Nexus, CTS, (Q)SAR Toolbox, EAWAG-BBD/PPS, and BioTransformer software. In terms of the number of predicted metabolites generated, the addition of GLORYx in our own analysis resulted in a greater number of compounds, likely due to the tool’s inclusion of low-probability metabolites.

Studying the totality of exposures in a truly–omics fashion is challenging for a variety of reasons: identifying all xenobiotics in a human biosample is analytically challenging, especially when there are chemicals with highly similarly structures and a complex exposure scenario. An additional challenge is the identification of all compounds–here, we found that a substantial number of metabolites would be simply invisible in most databases used for metabolite identification. The toxicity of many of them is simply unknown. While *in silico* tools can serve as a first step in identifying target compounds, a lack of publicly available or poorly resolved molecular structure data impedes the assessment of many commercially and toxicologically important chemicals.

The *in silico* prediction of xenobiotic metabolism using open-source tools is additionally constrained by molecular composition. For example, BioTransformer can accept single organic compounds (i.e., no mixtures) with a molecular weight below 1,500 Da^6^. GLORYx can predict metabolites for molecules containing 3 or more heavy atoms, so long as the compound is comprised of only C, N, S, O, H, F, Cl, Br, I, or P (de Bruyn Kops et al., 2021; Wishart et al., 2022). Moreover, neither method can predict reaction rates, and therefore the relative quantity of the potential metabolites remains unclear. Another major limitation in cheminformatics is the lack of specification between isomeric compounds. QSAR-ready SMILES strings have a lower resolution than isomeric smiles, and collapsing isomeric and isotopic information prohibits the assessment of these toxicologically important qualities. Despite these limitations, the models sufficiently captured empirical first-pass metabolites for the well-studied flame retardants BDE-47 and TBBPA.

It should be noted that many of the chemicals in this study have been phased out owing to human health or ecotoxicity concerns (for example, Mirex was banned in 1977). Yet because these chemicals are persistent, exposures can linger long after a flame retardant product is no longer in commerce. Mirex can remain in the food chain: in a biomonitoring study, blood samples obtained almost 20 years after Mirex was banned found higher levels in Intuit mothers vs. non-Inuit mothers in Arctic regions, presumably owing to different dietary patterns (Van Oostdam et al., 2004). PentaDBE–a mixture which includes BDE-47—was phased out in the mid-2000s, but owing to the long lifecycle of furniture and re-use of old furniture, it continues to be detected in house dust (Hammel et al., 2017).

Additionally, flame retardants are known to undergo abiotic and biotic degradation, which again increases the potential range of chemicals possible in a human biosample–to say nothing of the human microbiome. Although Biotransformer did predict a few chemicals as likely candidates for the human microbiome, this likely represents an understudied area. Although a few studies have looked at the effect of flame retardants on the human microbiome, and have suggested that microbial metabolites of flame retardants might mediate some observed effects, none so far have thoroughly examined the microbiome as a site of metabolism (Scoville et al., 2019). This is likely a further source of variation that has yet to be accounted for when looking at the health effects of flame retardants.

Flame retardants are often mentioned as cases of “regrettable substitutions”—when a compound with known adverse outcomes is swapped for a chemical which eventually proves as bad, if not worse, than the original chemical. Earlier flame retardants, such as PCBs, were phased out owing to their persistence, potential to bioaccumulate and ecotoxicity; they were replaced with PBDEs, which in turn were replaced OPEs–each of which have been associated with human health hazards. Lacking precise data to quantify exposures and hazards associated with each replacement, it is difficult to know the extent to which the substitution was an improvement or not, but the failure to have adequate data to answer this question definitively–especially when exposure to pregnant mothers and children is near ubiquitous–does speak to a significant lacuna that only a more comprehensive, exposomics based approach can fill.

## Data Availability

The original contributions presented in the study are included in the article/Supplementary Material, further inquiries can be directed to the corresponding author.
