# Supplementary figures and images for: Using in silico tools to predict flame retardant metabolites for more informative exposomics-based approaches
Source: Front Toxicol. 2023 Oct 16;5:1216802. doi: 10.3389/ftox.2023.1216802 (PMC10613991; doi:10.3389/ftox.2023.1216802)

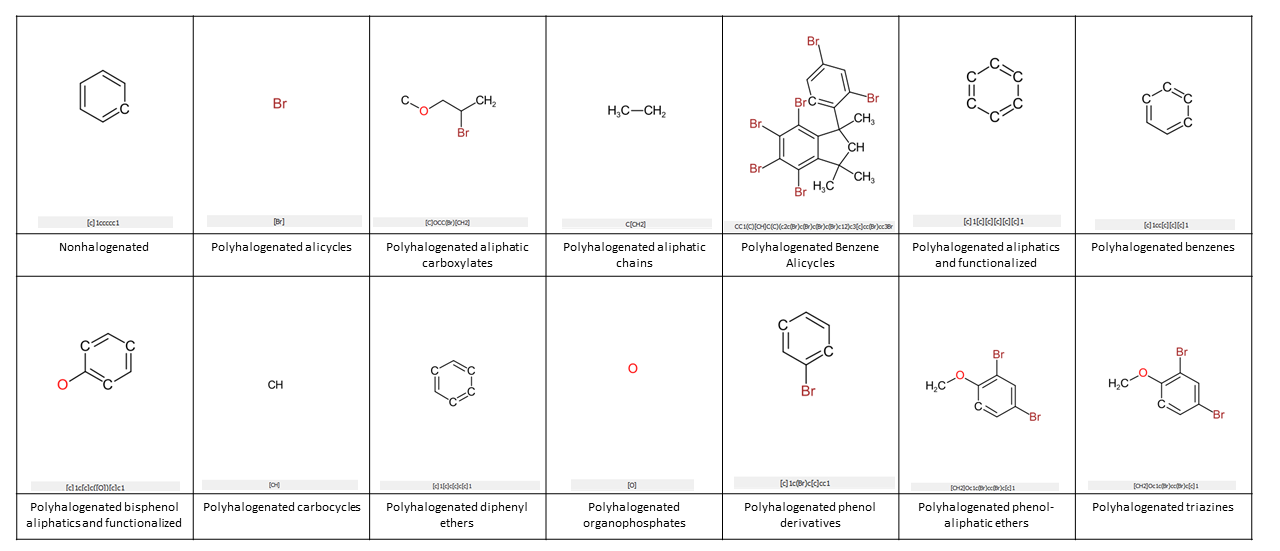

Supplement: Supplementary file 3 [file Image1.TIF]
